# Deep Sclerectomy with Nonabsorbable Implant (T-Flux) in Patients with Pseudoexfoliation Glaucoma

**DOI:** 10.1155/2017/6923208

**Published:** 2017-01-15

**Authors:** Pavel Studeny, Alina-Dana Baxant, Jana Vranova, Pavel Kuchynka, Jitka Pokorna

**Affiliations:** ^1^Department of Ophthalmology, University Hospital Kralovske Vinohrady, 3rd Faculty of Medicine, Charles University, Prague, Czech Republic; ^2^Department of Medical Biophysics and Informatics, 3rd Faculty of Medicine, Charles University, Prague, Czech Republic

## Abstract

*Purpose.* To evaluate the effectiveness of deep sclerectomy with T-flux implant (DS T-flux) in patients with pseudoexfoliation glaucoma (PExG).* Methods.* 20 eyes of 18 patients with medically uncontrolled PExG have undergone DS T-flux implantation. Postoperatively we evaluated the IOP values and the frequency of complications. The minimum follow-up time was 12 months (20 eyes) and the maximum 24 months (10 eyes).* Results.* The mean preoperative IOP was 36.8 ± 8.7 mmHg. The IOP significantly decreased throughout all postoperative periods (*P* < 0.05) and reached 1 day after surgery 11.45 ± 6.6 mmHg; 3 months 13.45 ± 3.6 mmHg; 12 months 14 ± 2.8 mmHg; and 24 months 14.80 ± 2.4 mmHg. Complete success rate, defined as IOP ≤ 18 mmHg without medication, was 85% (17/20 eyes) at 12 months. Qualified success rate, defined as IOP ≤ 18 mmHg with or without medication, was 100% (20/20 eyes). The most frequent postoperative complications were mild hyphaema (9 patients, 45%), choroidal detachment (3 patients, 15%), and hypotony—IOP < 5 mmHg (2 patients, 10%).* Conclusions.* DS with T-flux implant is a safe and effective surgical treatment method for medically uncontrolled PExG. The number of complications is low.

## 1. Introduction

Pseudoexfoliation syndrome (PEx) is an age-related systemic disease characterized by the production and accumulation of an abnormal fibrillar material as small, whitish deposits in many ocular and extraocular tissues [1–3]. It was first described by Lindberg in 1917 [4, 5]. The reported prevalence of PEx varies from 0.2% to 30% in different studies and populations; in European countries it is reported as being between 4 and 6.5% [6–16]. In many of these patients a special type of secondary glaucoma progressively develops—pseudoexfoliation glaucoma (PExG). The percentage of patients with PEx who develop secondary glaucoma varies in different studies. In one series of 100 consecutive subjects with PEx, glaucoma was detected in 7% and ocular hypertension in 15% of patients [17]. In the Jeng et al. study, 16% of patients with PEx initially had antiglaucoma therapy, and, in the remaining patients, the probability of being placed on therapy was 44% at 15 years [18]. In the Grodum study, after a mean of 8.7 years, 54 out of 98 patients (55.1%) with PEx had developed glaucoma [19].

PExG is thought to arise secondarily to congestion of the trabecular meshwork by pseudoexfoliative material. It is the most common form of secondary open-angle glaucoma. In general, PExG in comparison with primary open-angle glaucoma (POAG) has a greater resistance to medicamentous treatment, rapid progression, higher diurnal intraocular pressure (IOP), fluctuation, and poor prognosis [19–22].

If medical treatment is not sufficient to manage PExG, subsequent interventions are surgical, typically comprising laser trabeculoplasty, trabeculectomy, and the implantation of a glaucoma drainage device [20].

Deep sclerectomy (DS) is a nonpenetrating filtration procedure for the surgical treatment of medically uncontrolled open-angle glaucoma. It was designed in an attempt to lower the risk of the incidence of some postoperative complications after penetrating glaucoma surgery and thus offers both surgeons and patients a safer, more convenient option [23–25].

An evaluation of the effectiveness of deep sclerectomy and nonabsorbable glaucoma implants in patients with PExG is the main aim of this work.

## 2. Materials and Methods

This was a prospective, consecutive, interventional case series including a total of 20 eyes of 18 patients with pseudoexfoliation glaucoma. The patients selected were all medically uncontrolled with maximal medical therapy. Uncontrolled glaucoma was defined as uncontrolled intraocular pressure (>21 mmHg) under maximal tolerable medical treatment and with documented progression of visual field defects and optic nerve morphology. Therefore, the surgical procedure DS with T-flux was performed. Surgeries were carried out in the period from 9/2011 to 9/2013. Of those 18 patients, 11 were men and 7 women; mean age was 69.9 ± 9.04 years (min 58, max 87). Eight eyes were pseudophakic and 2 eyes had previous classic trabeculectomy. Informed consent was obtained from all the patients. Institutional ethical review board approval was obtained for the procedures and the tenets of the Helsinki Declaration were followed.

All the surgical procedures were performed by 1 surgeon (Pavel Studeny). The technique of deep sclerectomy has been described previously (Fyodorov 1989 [23], Kozlov et al. 1990 [24], Sanchez et al. 1997 [26], and Shaarawy et al. 2004 [25]). The procedures were carried out under subconjunctival anaesthesia; the conjunctiva was opened in the fornix. After deep sclerectomy Schlemm's canal endothelium was peeled away by forceps. A nonabsorbable T-flux implant (Carl Zeiss Meditec, Wetzlar, Germany) was placed underneath the flap. The two arm extremities of the implant were anchored into Schlemm's canal. We did not use sutures for fixation of the implant. None of the patients received mitomycin during the surgery or 5-Fluorouracil in the postoperative period. The scleral flap was closed with two 8/0 absorbable sutures and the conjunctiva was closed with one continuous absorbable suture 8/0.

Preoperatively and at all postoperative visits, the patients had a complete ocular examination. After surgery we evaluated the IOP measured with a Goldmann applanation tonometer on the first and seventh day as well as in 1, 3, 6, 9, 12, 15, 18, 21, 24, 27, and 30 months. The required antiglaucoma medication and all postoperative complications were noted. Complications were defined as follows: hypotony was defined as a postoperative IOP < 5 mmHg; hyphaema was considered present when erythrocytes were seen in the anterior chamber. The anterior chamber was considered shallow when there was an iridocorneal touch in the periphery. Choroidal detachment was considered present when seen in the peripheral retina using fundus biomicroscopy. Surgery-related cataract was defined by a rapid decrease (over a period of 1 month) in visual acuity and the development of cortical opacity. The minimum follow-up time was 12 months and 10 eyes had a follow-up time of 24 months.

Preoperative data versus postoperative data were analysed using the paired *t*-test. Statistical measurements are the mean ± standard deviation and significant *P* values were less than 0.05. Statistical analysis was performed using SPSS statistic software, version 15.0, for Windows (SPSS, Inc., IL, US).

## 3. Results

**Figure 1:**
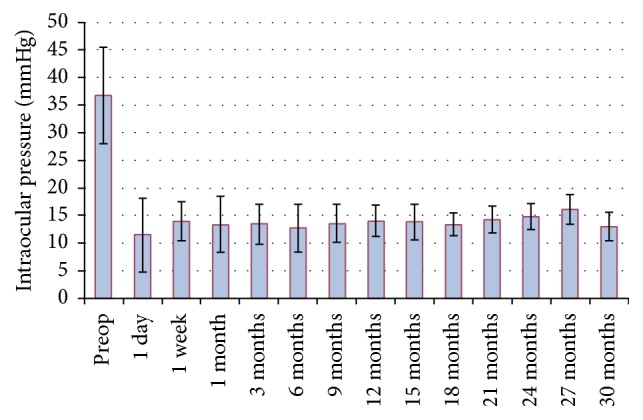
The mean intraocular pressure after deep sclerectomy with T-flux.

**Figure 2:**
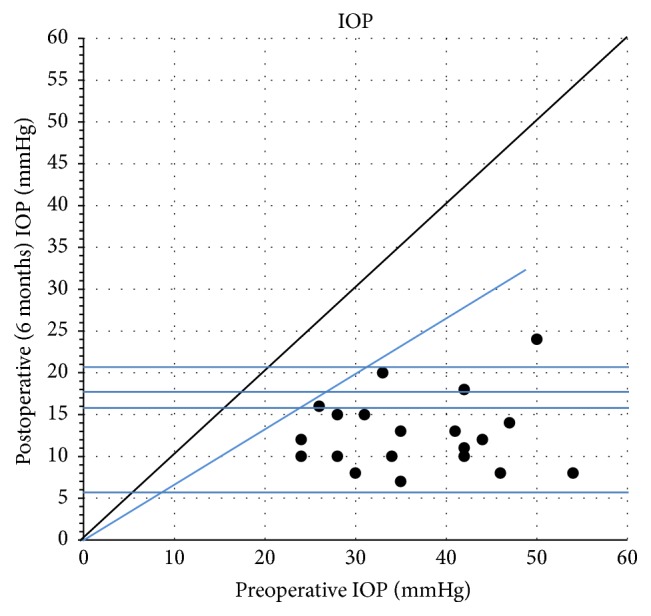
Average IOP preoperatively versus six months postoperatively.

The mean preoperative IOP was 36.8 ± 8.7 (min 24, max 54) mmHg. Preoperatively, the patients received 2–4 glaucoma medications (local as well as systemic), on average 3.3 ± 0.56.  Table 1 summarises the postoperative IOP. The mean IOP on the first postoperative day was 11.45 ± 6.6 mmHg and at 3 months was 13.45 versus 36.8 mmHg. At 6 months after surgery IOP was reduced by 65.5%, from 36.8 to 12.7 mmHg (Figure 2). Furthermore at 12 months IOP was reduced by 14.0 versus 36.8 mmHg and at 24 months by 60% (14.8 versus 36.8 mmHg). In all reporting periods the decrease in IOP in comparison with the preoperative value was statistically significant (Table 1). Postoperative intraocular pressure remained stable over the follow-up period (Figure 1). In three patients in the postoperative period (1 month after surgery) we noticed a slight elevation in IOP (above 25 mmHg); therefore goniopuncture was performed. Out of three patients the values normalized without antiglaucoma therapy in one patient, while long-term monotherapy was recommended for two patients. The complete success rate, defined as IOP 18 mmHg or lower than 18 without medication, was 85% (17/20 eyes) at 12 months. The qualified success rate, defined as IOP 18 mmHg or lower than 18 mmHg with or without medication, was 100% (20/20 eyes).

BCVA was assessed using the ETDRS eye charts. On the first postoperative day BCVA was decreased depending on the complications which occurred. The worst BCVA (hand movement) was noticed in 3 eyes, in which there were a larger amount of blood in the anterior chamber and choroidal detachment. The best BCVA on the first day after surgery was the same as before surgery (in 8 eyes).

Half a year after surgery visual acuity (BCVA) stabilised in all studied eyes. Six months after surgery the average visual acuity loss was 0.55 letters on the ETDRS chart. At six months postoperatively versus the period prior to surgery: 11 eyes had the same visual acuity; 1 eye had a loss of 10 letters on the ETDRS chart; 3 eyes had a loss of 2 letters; 2 eyes had a loss of 3 letters; 1 eye gained one letter and 2 eyes had a gain of 5 letters on the ETDRS chart.

During surgery we noticed on one occasion a small perforation of trabeculo-Descemet membrane, without iris prolapse, so the surgery was finished without conversion.

The most frequent complication after surgery was mild hyphaema; the blood from the anterior chamber was fully absorbed within 1 week in all cases. This complication was seen in 9 patients (45%). On three occasions (15%) we noticed choroidal detachment, which completely reattached within 1 month in all three patients. On two occasions (10%) the intraocular pressure on the first day after surgery was lower than 5 mmHg (hypotony), while 1 week after surgery no patient had IOP lower than 5 mmHg. There has been one case of leak as a result of conjunctival suture loose. There were three cases of goniopuncture performed due to the IOP elevation above 25 mmHg.

No shallow anterior chamber, no surgery-induced cataract, and no bleb-related endophthalmitis were observed in our group of patients.

## 4. Discussion

Deep sclerectomy is preferred by many authors in the therapy of medically uncontrolled primary open-angle glaucoma prior to classical trabeculectomy. The main advantage of nonpenetrating glaucoma surgery is the reduced number of intraoperative and postoperative complications [27–31]. This is due to the fact that there is no direct communication with the anterior chamber, thereby potentially reducing the risk of infection. Another advantage is that there is no significant fluctuation of intraocular pressure during surgery. Therefore, complications such as hyphaema [29], choroidal bleeding, and loss of the rest of the visual field [32] are theoretically less likely.

On the other hand, some authors have described the lower efficiency of nonperforating operations in comparison with trabeculectomy, especially in long-term follow-up time [28, 31, 33]. However, the use of implants significantly increased the long-term effectiveness of the operations [34–36].

Despite the advantages that DS with T-flux implantation have in patients with primary glaucoma, its use in patients with secondary glaucoma remains slightly controversial. In patients with pseudoexfoliation syndrome there occurs a pathological deposition of material on the internal structures of the anterior chamber, in the iridocorneal angle, and in the trabecular meshwork (TM). This is considered to be the main cause of intraocular pressure elevation. Material deposited on the inner side of the TM remains in place during nonpenetrating surgery. Nevertheless, many authors describe the successful use of deep sclerectomy in patients with this type of secondary glaucoma [37–40]. We were able to observe very good filtration of aqueous humor through the TM during surgery in our patients with pseudoexfoliation glaucoma.

We consider the peeling of the endothelium of Schlemm's canal very important.

It is typically very strongly pigmented in patients with PExG.

Mendrinos et al. [37] described the results of deep sclerectomy with collagen implant in patients with exfoliative glaucoma. After a mean follow-up time of 48.5 ± 12.2 months, mean IOP was significantly reduced from 29.9 ± 8.1 mmHg preoperatively to 13.2 ± 3.2 mmHg [37].

Ollikainen et al. [38] compared the results of mitomycin C augmented deep sclerectomy in patients with primary open-angle glaucoma and exfoliation glaucoma. One year after surgery the results in both groups were very similar. The mean IOP decreased from 25.4 ± 8.3 mmHg to 11.2 ± 5.6 mmHg in the group of patients with exfoliation glaucoma, and 75.7% of surgeries were a complete success. They noticed only a few postoperative complications, although choroidal detachment occurred in 10.8% of eyes [38].

Rekonen et al. [41] compared the results of deep sclerectomy with implant (collagen or hyaluronate) in patients with primary open-angle and exfoliation glaucoma. At 18 months, complete success had been achieved in 44.9% and qualified success in 71.6% of patients with PExG. The mean IOP was 16.3 mmHg in this group of patients. Reoperations were required in seven (18%) PExG eyes [41].

Drolsum [39] compared the results of deep sclerectomy with implant (collagen or T-flux) in patients with POAG and PExG. In his study, PExG patients had significantly higher success rates over time than POAG patients. After a mean follow-up of 19.9 ± 10.9 months, complete success (IOP below 19 mmHg without therapy) was seen in 60.7% in the group of PExG patients. Levelled hyphaema occurred in 35.7% after surgery in this group [39].

Although preoperative values of IOP in our group were higher than in the above-mentioned works (36.8 ± 8.7 mmHg), we achieved similar postoperative values. The results in our group of patients were comparable to or often better than the above-mentioned studies. One year after surgery we saw a complete success rate in 85% and qualified success in 100%. In neither case was reoperation required. The number of preoperative and postoperative complications as in the previous work was also low. However, the major limitation of our study is the relatively small number of patients and short follow-up period of 12 (or 24) months.

The reason for the high success rate in our group of patients is likely to be the use of nonabsorbable T-flux implant and very careful peeling of Schlemm's canal. Compared with our own previously published results of this type of surgery in patients with POAG, we confirm higher efficiency in patients with PExG [42]. This is consistent with the conclusion by Drolsum [40].

## 5. Conclusions

Deep sclerectomy with implantation of T-flux is a safe and effective method for the treatment of patients with medically uncontrolled PExG. The number of intraoperative and postoperative complications is low.

## Figures and Tables

**Table 1:** Statistical significance of postoperative intraocular pressure reducing.

	Number	Median IOP (mmHg) ± SD	Min	Max	*P* value
Preoperatively	20	36.8 ± 8.7	24	54	<0.001
1 day	20	11.45 ± 6.6	3	16	<0.001
7 days	20	13.95 ± 3.5	7	18	<0.001
1 month	20	13.35 ± 5.0	8	27	<0.001
3 months	20	13.45 ± 3.6	8	20	<0.001
6 months	20	12.70 ± 4.3	7	24	<0.001
12 months	20	14.00 ± 2.8	10	19	<0.001
18 months	10	13.40 ± 2.1	18	21	<0.001
24 months	10	14.80 ± 2.4	10	16	<0.001
